# p62 Suppressed VK3-induced Oxidative Damage Through Keap1/Nrf2 Pathway In Human Ovarian Cancer Cells

**DOI:** 10.7150/jca.34423

**Published:** 2020-01-01

**Authors:** Mei-hui Xia, Xiao-yu Yan, Lei Zhou, Long Xu, Li-chao Zhang, Hao-wei Yi, Jing Su

**Affiliations:** 1 Department of Obstetrics, the First Bethune Hospital of Jilin University, Changchun, Jilin, China.; 2 Department of Pathophysiology, Key Laboratory of Pathobiology, Ministry of Education, College of Basic Medical Sciences, Jilin University, Changchun, Jilin, China.; 3 Department of Pathology, Affiliated Hospital to Changchun University of Chinese Medicine, Changchun 130021, China.

**Keywords:** p62, VK3, ROS, Drug resistance, Nrf2, Keap1

## Abstract

Imbalance of redox homeostasis may be responsible for the resistance of cancer to chemotherapy. Currently, increasing studies demonstrated that vitamin K3 (VK3), which promoted the production of ROS, had potential to be developed as an anti-tumor agent. We found SKOV3/DDP cells with high levels of p62 were insensitive to VK3 compared with SKOV3 cells. Furthermore, Nrf2 downstream antioxidant genes such as HO-1(heme oxygenase 1) and NQO1 (NAD (P) H: quinone oxidoreductase 1) were upregulated in SKOV3/DDP cells with VK3 treatment, which indicated VK3 activated Nrf2 signaling in SKOV3/DDP cells. Moreover, co-localization of p62 and Keap1 was also observed. Suppression of p62 expression increased the apoptosis induced by VK3, and the expression of Nrf2, HO-1 and NQO1 were all downregulated in SKOV3/DDP cells. Our results suggested that overexpressed p62 may protect cells from oxidative damage caused by VK3 through activating Keap1/Nrf2 signaling in ovarian cancer.

## Introduction

Many commonly used anti-tumor drugs induced apoptosis of cancer cells by producing reactive oxygen species (ROS), such as vinblastine, cisplatin, mitomycin C, doxorubicin, camptothecin and inostamycin 1. However, cancer cells may activate strong antioxidant signaling against the damage caused by these chemotherapeutic agents 2. Current studies suggested that high levels of antioxidant genes such as gamma-glutamyl-transpeptidase, glutathione-S-transferase, glutathione peroxidase 3, 4 and heme oxygenase 1 (HO-1) 5 may be involved in tumor chemotherapeutic resistant mechanism.

Nucleus factor erythroid-2 related factor 2 (Nrf2) is a critical transcription factor regulating the transcription of anti-oxidative genes encoding for various detoxifying enzymes and antioxidant proteins 6. Currently, studies found Kelch-like ECH-associated protein 1 (Keap1), an adaptor protein for the Cullin3-based E3 ubiquitin ligase, binds and targets Nrf2 for degradation 7-9. However, oxidative stress induces modification of cysteine residue in Keap1 10, which allows Nrf2 to dissociate from Keap1 complex and increases the translocation of nucleus. Notably, aberrant activation of the Keap1-Nrf2 pathway has been detected in various types of cancer cells that is resistant to chemotherapy 11, 12. However, the regulatory mechanism of Keap1-Nrf2 pathway in ovarian cancer is still unclear 13-15.

p62 works as a multifunctional protein that participates in the autophagy regulation and several cellular signaling pathways such as redox signaling, inflammation, proliferation and apoptosis 16-18. Increasing studies have shown that overexpression of p62 is positively correlated with the malignancy of breast and prostate cancers 7, 19. Our previous studies revealed that p62 was highly expressed in SKOV3/DDP cells and was involved in cisplatin resistance by promoting ubiquitinated proteins degradation in ovarian cancer 20. Recently, it has been proved that p62 inhibits Nrf2 degradation by disrupting the Keap1-Nrf2 interaction 21-23, which leads to Nrf2 stabilization and nucleus translocation. These findings suggest that p62 may participate in chemotherapy resistance through regulating Nrf2-related anti-oxidative signals.

Vitamin K3 (VK3) was reported to work as an anti-tumor agent by producing ROS, including hydroxyl radicals, superoxide radicals, and hydrogen peroxide 24. In this study, we explored the role of p62 in VK3 induced oxidative damage and revealed the possible resistant mechanism of chemotherapy targeting redox balance in ovarian cancer cells.

## Materials and methods

### Cell lines

SKOV3 cells and SKOV3/DDP cells were purchased from the Chinese Academy of Medical Sciences and Peking Union Medical College. They were cultivated in a humidified atmosphere with 5% CO2 at 37 °C in complete RPMI 1640 medium (Gibco, Carlsbad, CA, USA). To maintain drug resistance, SKOV3/DDP cell were cultured with 1 μg/ml cisplatin (Sigma-Aldrich, St. Louis, MO, USA).

### MTT assay

The density of plated cells is 1×10^4^ cells each well. Cells were then added different concentrations of VK3 and cultivated for 16 h. MTT solution (2 mg/ml) treated to each well were incubated for 4 h. After discard the MTT solution and change it to dimethyl sulfoxide (100 μl/well), we use the Vmax Microplate Reader to observe the value of OD at 570 nm (Molecular Devices, Sunnyvale, CA, USA).

### Flow cytometry

Annexin V-FITC / PI were used to stain cells (KeyGEN Biotech, Nanjing, China) according to the manufacturer's protocol. FACScan flow cytometer has been used to detect the rate of apoptosis (Becton Dickinson, Franklin Lakes, NJ, USA).

### Western blotting

Cells were harvested and lysed in RIPA buffer. Lysates were incubated at 4 °C for 45 min. After centrifuging, the Protein Assay Kit has been used to determine the concentration (Bio-Rad, Hercules, CA, USA). Then samples were electrophoresis through 12% (w/v) SDS polyacrylamide gel. Subsequently, we transferred the proteins onto PVDF membranes (Millipore, Bedford, MA, USA). After washing with TPBS buffer three times, membranes were incubated with target antibodies and secondary antibody (Thermo, Waltham, MA, USA). Method of coloration diaminobenzidine (Sigma-Aldrich) and Quantity One software (Bio-Rad) were used 25. Antibodies used in this study were anti-p62 and caspase 3 (Abcam, Cambridge, MA, USA ), anti-β actin and anti-Keap1 (Proteintech, Chicago, IL, USA), anti-Nrf2 (ABclonal Biotechnology Co., Ltd.) and anti-Lamin A/C (Santa Cruz Biotechnology, Santa Cruz, CA, USA).

### Measurement of ROS generation

We used 2',7'-dichlorofluorescein diacetate (DCFH-DA) (Beyotime, Hangzhou, China) to estimate the intracellular generation of ROS. Cells were treated with VK3 (15 μM) for 8 or 16 h. Both two cells were washed by PBS followed with 50 μM DCFH-DA treatment. Then cells were observed by microscopy. At the same time, cells in another plate were harvested and FACScan flow cytometer has been used to measure the fluorescence intensity (Becton Dickinson).

### Preparation of nucleus extracts

Cells were harvested and lysed in homogenization buffer and then centrifuged as described before 26. We discarded the supernatant and the precipitate was resuspended in RIPA buffer (Beyotime) according to the instructions.

### RNA extraction and RT-PCR

Total RNA was separated from cells using Trizol reagent (Invitrogen). cDNAs were generated from RNA samples by reverse transcription. Primers for HO-1 and NQO1 were described previously 26; HO-1 primers were 5′-CAGAAGAGCTGCACCGCAAG-′3 and 5′-GGTAGAGCTGCTTGAACTTG-3′, and NQO1 primers were 5′-GATATTGTGGCTGAACAA-3′ and 5′-TGCTATATGTCAGTTGAG-3′. GAPDH was served as the normalization control; primers for GAPDH were 5′-GGGTGATGCTGGTGC TGAGTATGT-3′ and 5′-AAGAATGGGAGTTGCTGTTGAAGT-3′. Then we electrophoresed the PCR products and GIS 1D gel image system software were used to analyze (Tanon, Shanghai, China).

### p62 RNAi

Transfections of si-p62 and si-scrambled was described previously 20. Briefly, cells were transfected using Lipofectamine 2000 in six-well plates (Invitrogen). Cells were subjected to experimental analyses 2 days after transfection.

### Immunoprecipitation

Cells were harvested and lysed in IP-lysis buffer (Beyotime) for 45 min. After centrifuged at 12,000 g for 10 min the lysates were transferred to another tube containing Agarose-Protein A/G (Santa Cruz) and 1 μg Keap1 antibody. Incubating overnight with gentle rotation, the mixture was centrifuged at 5000 g for 30s. The Agarose-Protein A/G was washed and immunoprecipitated proteins were eluted by boiling in IP-lysis buffer prior to western blotting analysis.

### Immunofluorescence

After treated with VK3 treatment, cells were fixed with 4% PFA for 20 min. Cells were then incubated with primary antibodies anti-p62 and anti-Keap1 (both at 1:100 dilution in PBS) overnight at 4°C. After washing with PBS three times, the cells were then treated with appropriate secondary antibodies. Confocal microscopy was used for observation.

### Statistical analysis

Data were analyzed using a two-tailed Student t-test. A value of P < 0.05 was considered statistically significant. And experiments have been repeated three times independent.

## Results

### VK3 promoted the apoptosis of SKOV3 ovarian cancer cells

**Figure 1:**
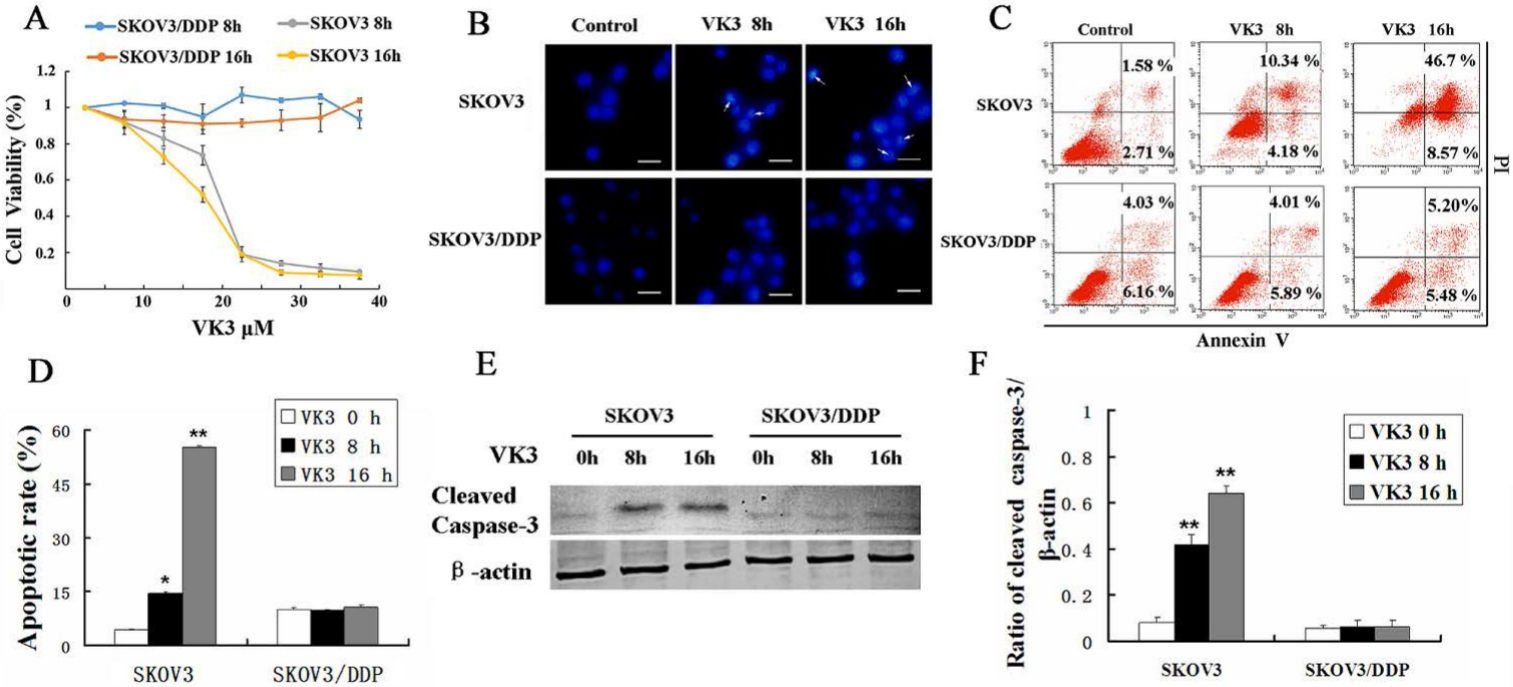
** VK3 promoted the apoptosis of SKOV3 ovarian cancer cells. (A)** SKOV3 and SKOV3/DDP cells were treated with VK3 for 8 and 16 h. The MTT assay was used to examine the cell viability. Data are presented as mean ± SD, n = 3. **(B)** Both cells were treated with 15 µM VK3 and stained with Hoechst 33342. Cell morphology was observed by fluorescence microscopy. Arrows indicate apoptotic cells. Scale bar = 20 µm. (C) Cells were stained with Annexin V-FITC/PI, and the ratio of apoptosis was detected using a FACScan flow cytometer. Data are presented as mean ± SD, n = 3. **(D)** Apoptotic rate in **(C)** was quantified in both cells. Data are presented as mean ± SD, n = 3. *P < 0.05 and **P < 0.01 compared with untreated cells. **(E)** Western blotting was used to analyze the expression of cleaved caspase-3. **(F)** The expression of cleaved caspase-3 was quantified. Data are presented as mean ± SD, n = 3. **P < 0.01 compared with untreated cells.

VK3 has been reported to inhibit proliferation and induce apoptosis in cancer cells 27, 28. MTT assay showed that VK3 significantly inhibited the cell viability in SKOV3 cells compared with SKOV3/DDP cells and especially in high dose (inhibition rate is 80% in 20μM in SKOV3 cells) (Fig. 1A). Then we investigated whether VK3 could cause apoptosis through Hoechst 33342 staining. In SKOV3 cells, the obvious apoptotic chromatin condensation was observed after treatment with VK3 for 8 and 16 h (Fig. 1B). Annexin V-FITC/ PI staining showed that 14.52% of SKOV3 cells initiated apoptosis in response to VK3 treatment for 8h and 55.27% for 16h (Fig. 1C and 1D). Moreover, western blotting showed that the expression of cleaved caspase-3 was higher in VK3-treated SKOV3 cells compared with VK3-treated SKOV3/DDP cells (Fig. 1E and 1F). Together, our results demonstrate that VK3 inhibits proliferation and induces apoptosis in SKOV3 ovarian cells.

### VK3 induced apoptosis in SKOV3 cells through increasing generation of ROS

**Figure 2:**
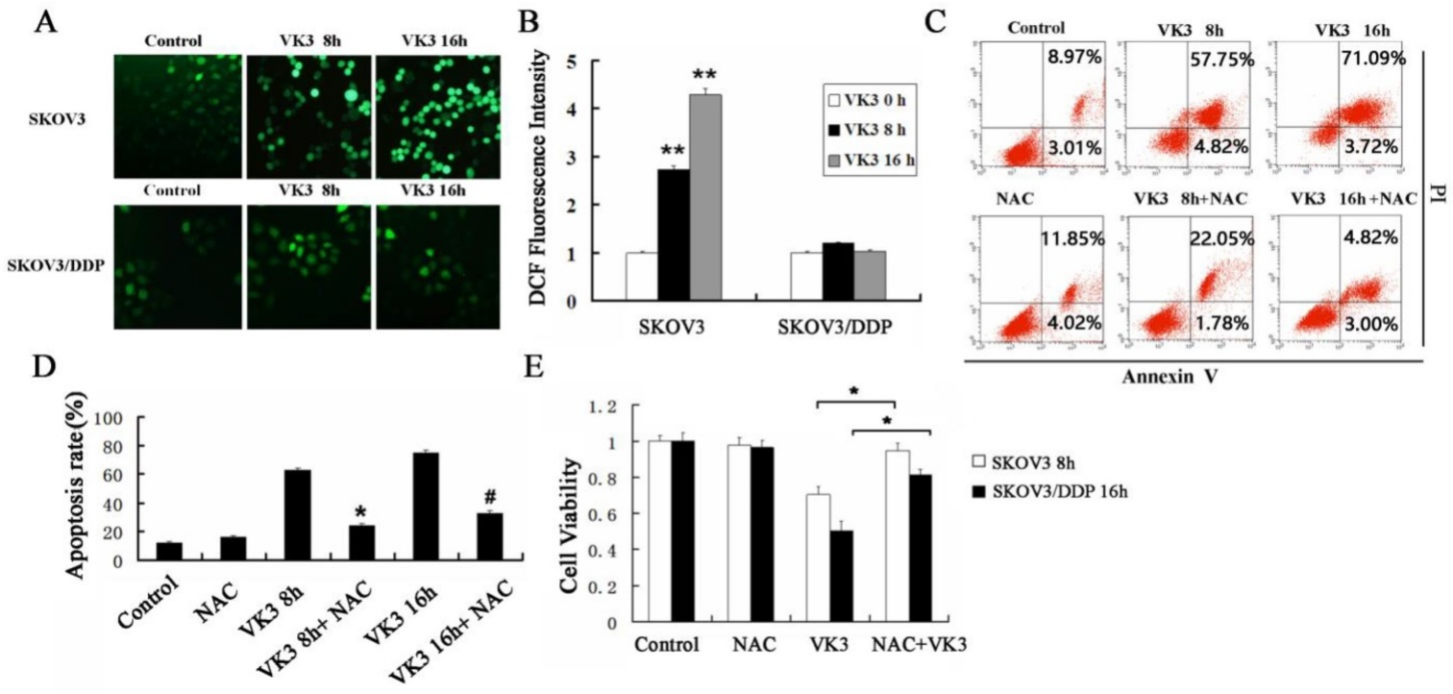
** Inhibition of ROS reduces VK3-induced cell death in ovarian cancer cells. (A)** Both cells were treated with VK3 (15 µM) for 8 or 16 h and ROS generation was determined using 50 µM DCFH-DA. DCF fluorescence intensity was detected by fluorescence microscopy (100×). **(B)** Quantification of DCF fluorescence intensity in (A). Data are presented as mean ± SD, n = 3. **P < 0.01 compared with control. **(C)** SKOV3 cells pretreated with 40 μM NAC for 1h were stained with Annexin V-FITC/PI. FACScan was used to count positively stained cells. **(D)** Quantitation of apoptotic rate in SKOV3 cells in (C). Data are presented as mean ± SD, n = 3. *P < 0.05 compared with 8 h VK3 treatment; **^#^***P* < 0.05 compared with 16 h VK3 treatment. **(E)** The MTT assay was used to examine the cell viability with 40 µM NAC pretreatment followed by 15 µM VK3 culture. Data are presented as mean ± SD, n = 3. *P < 0.05 compared with VK3 treatment alone.

Previously, the antitumor effect of VK3 has been shown to be due to the production of ROS by redox cycling 29. We next examined the level of ROS through DCFH-DA assay. The results showed that VK3 caused high levels of ROS in SKOV3 cells, while ROS levels did not change significantly in SKOV3/DDP cells (Fig. 2A and 2B). NAC (antioxidant N-acetylcysteine) was commonly used to inhibit ROS. In the next part we used NAC as ROS inhibitor to further confirm the role of ROS in VK3-induced apoptosis. According to results of Annexin V/PI assay, the apoptotic rate was 23.83% and 32.53% with NAC pre-treatment in SKOV3 cells, which were decreased compared to the cells exposed to VK3 (Fig. 2C and 2D). Furthermore, MTT assay results showed that NAC pre-treatment also attenuated the VK3-induced inhibition of SKOV3 cell viability (Fig. 2E). These findings indicated that the increase of ROS induced by VK3 may be involved in the cell viability and apoptotic response of SKOV3 cells.

### VK3 activated the Nrf2 signaling in SKOV3/DDP ovarian cancer cells

**Figure 3:**
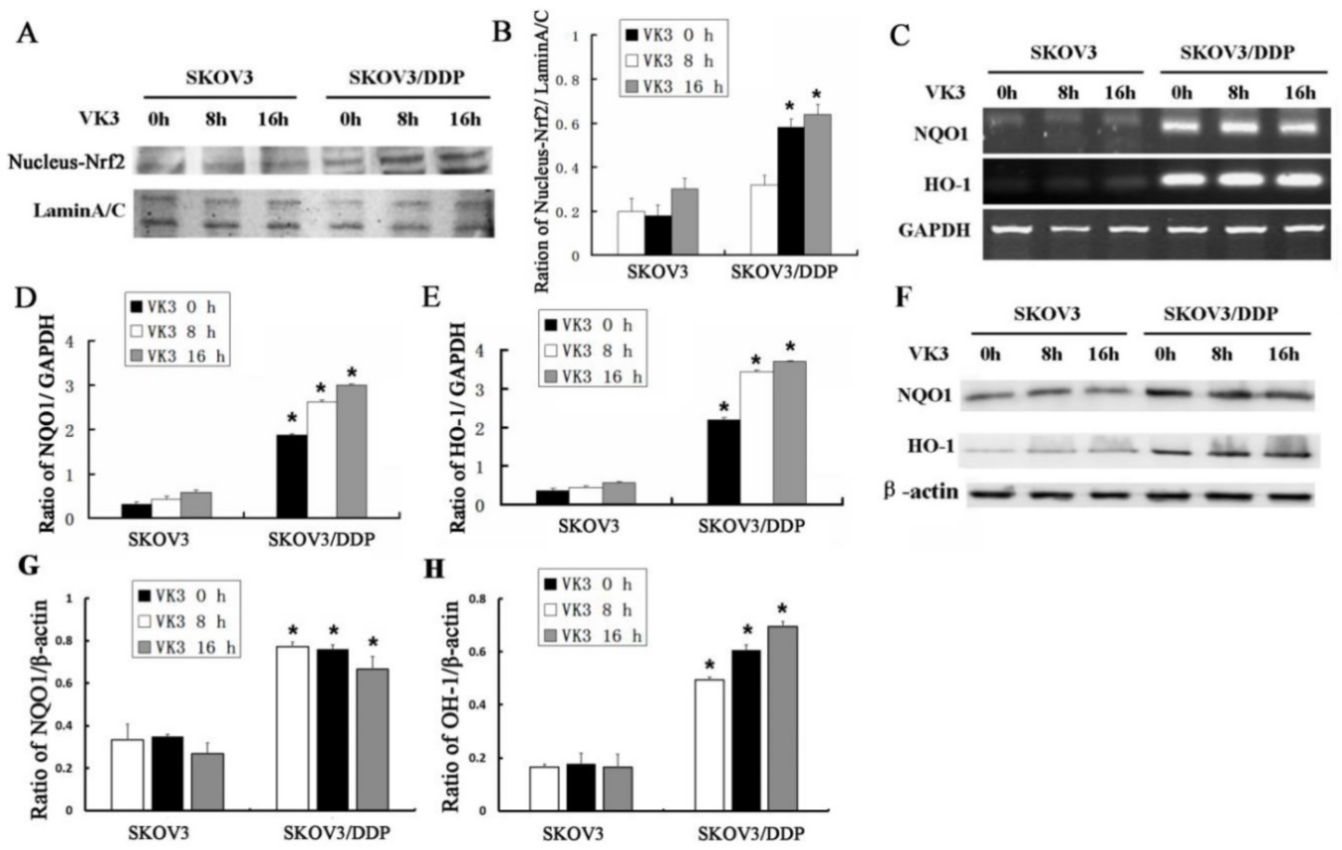
** VK3 activates the Nrf2 pathway in SKOV3/DDP cells. (A)** Both cells were treated as before. Nucleus extracts were subjected to immunoblot analysis with anti-Nrf2 and anti-LaminA/C. **(B)** Quantitation of nucleus Nrf2 protein level in (A). Data are presented as mean ± SD, n = 3. *P < 0.05 compared with untreated cells. **(C)** Total RNAs were prepared and NQO-1 and HO-1 mRNA levels were analyzed by RT-PCR. **(D, E)** Quantitation of HO-1 and NQO1 levels in (C). Data are presented as mean ± SD, n = 3. *P < 0.05 compared with SKOV3 cells. **(F)** The expression of HO-1 and NQO1 were examined by western blotting. **(G, H)** Quantitation of HO-1 and NQO1 levels in (E).Data are presented as mean ± SD, n = 3. *P < 0.05 compared with SKOV3 cells.

Nrf2 is a critical transcription factor that regulates genes encoding the anti-oxidative enzymes through antioxidant response elements in their promoter sequences 10, 11. To further elucidate the anti-oxidative mechanism in SKOV3 and SKOV3/DDP cells, we examined the expression of Nrf2 in nucleus through western blotting. Results showed that VK3 obviously increased the nucleus expression of Nrf2 in SKOV3/DDP cells (Fig. 3A and 3B). Nrf2 downstream genes NQO-1 and HO-1 were also overexpressed in SKOV3/DDP cells not only in mRNA but in protein levels in response to VK3 treatment (Fig. 3C-H). These results suggested that the up-regulation of Nrf2 pathway may be involved in VK3 resistant mechanism in ovarian cancer cells.

### Downregulated p62 inhibited the activation of Nrf2

**Figure 4:**
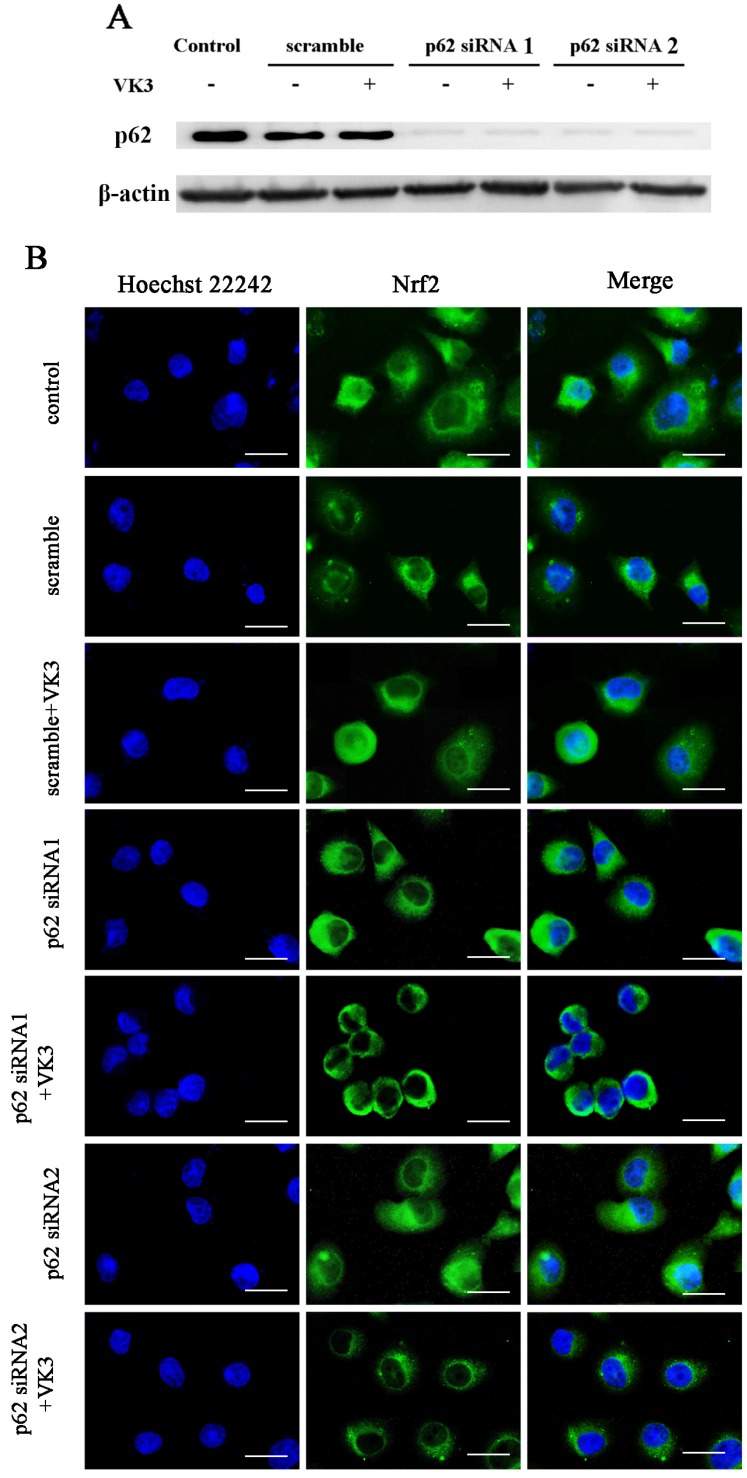
** Knockdown of p62 inhibited the translocation of Nrf2 in SKOV3/DDP cells with VK3 treatment. (A)** si-p62 or si-scrambled were transfected with SKOV3/DDP cells. After treated with 15 µM VK3 for 16h, cell lysates were subjected to immunoblot analysis **(B)** Cells were treated as (A), Immunofluorescence was performed with anti-Nrf2 antibodies and detected by fluorescence microscopy (scale bar, 25 µm).

Our previous study indicated that p62 was overexpressed in SKOV3/DDP cells and the high level of p62 was involved in cisplatin resistant mechanism through clearing ubiquitinated proteins in ovarian cancer cells 20. Furthermore, recent studies supported that p62 may also participate in the regulation of Nrf2 signaling 22, 23. In order to further confirm the role of p62 in Nrf2 signaling regulation, we inhibited p62 expression by RNAi in SKOV3/DDP cells and analyzed the translocation of Nrf2 to the nucleus as a result of VK3 treatment (Fig. 4A). The results showed that VK3 promoted Nrf2 nucleus translocation. However, the expression of Nrf2 in nucleus was decreased while p62 expression was suppressed (Fig. 4B). These results revealed that high level of p62 in SKOV3/DDP cells promoted the activation of Nrf2 in response to VK3 treatment.

### Inhibition of p62 increased apoptosis induced by VK3 in SKOV3/DDP ovarian cancer cells

**Figure 5:**
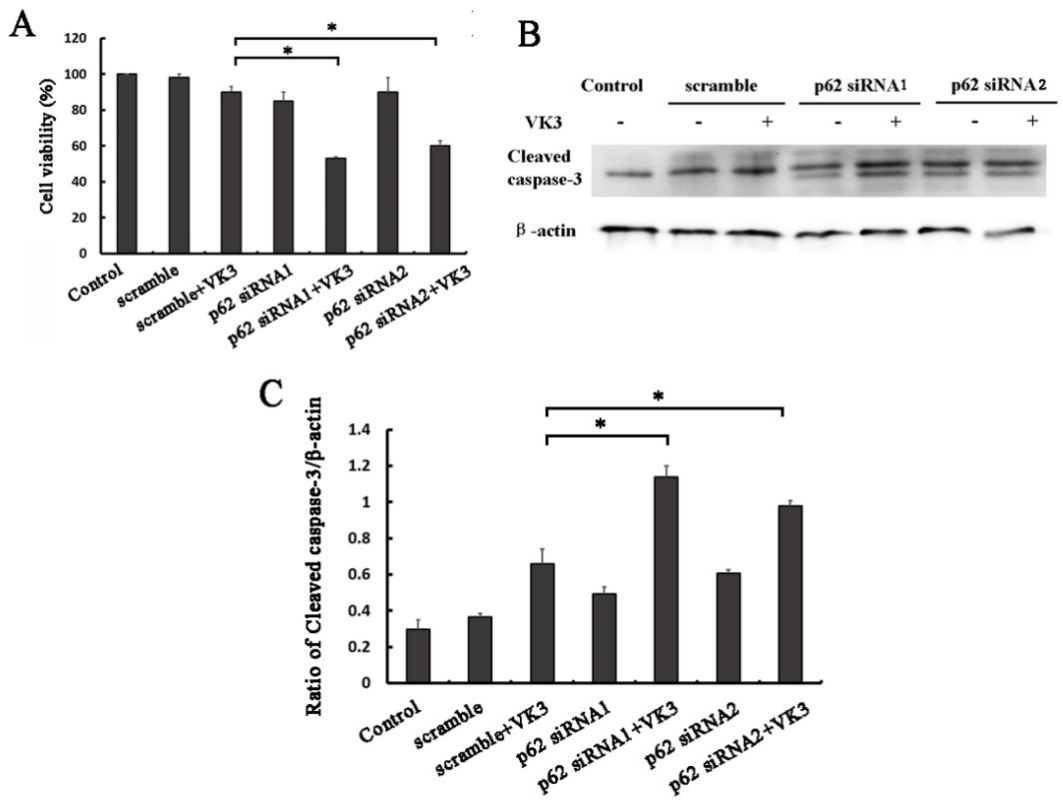
** p62 inhibition increased VK3-induced apoptosis in SKOV3/DDP cells. (A)** SKOV3/DDP cells were transfected with p62 or control siRNA. After treatment with 15 µM VK3 for 16h, MTT assays was used to evaluate cell viability. Data are presented as mean ± SD, n = 3. *P < 0.05 compared with si-scrambled + VK3 treatment group. **(B)** SKOV3/DDP cells were treated as (A). And the expression of cleaved caspase-3 was analyzed by Western blotting. **(C)** The expression of cleaved caspase-3 in (B) was quantified. Data are presented as mean ± SD, n = 3. *P < 0.05 compared with si-scrambled + VK3 treatment group.

In order to examine whether p62 mediated Nrf2 activation affected the response to VK3, we next performed MTT assays in SKOV3/DDP ovarian cancer cells with p62 RNAi. Consistent with our assumptions, the results proved that p62 inhibition decreased the cell viability compared with scramble group following VK3 treatment (Fig. 5A). Furthermore, the expression of cleaved caspase-3 was also increased in p62 silenced SKOV3/DDP cells (Fig. 5B and 5C). These results demonstrate that p62 inhibition enhanced the damage caused by VK3 treatment in SKOV3/DDP cells. Inactivation of Nrf2 through p62 downregulation increased cell sensitivity to VK3.

### p62 regulated Nrf2 by competitively binding with Keap1

**Figure 6:**
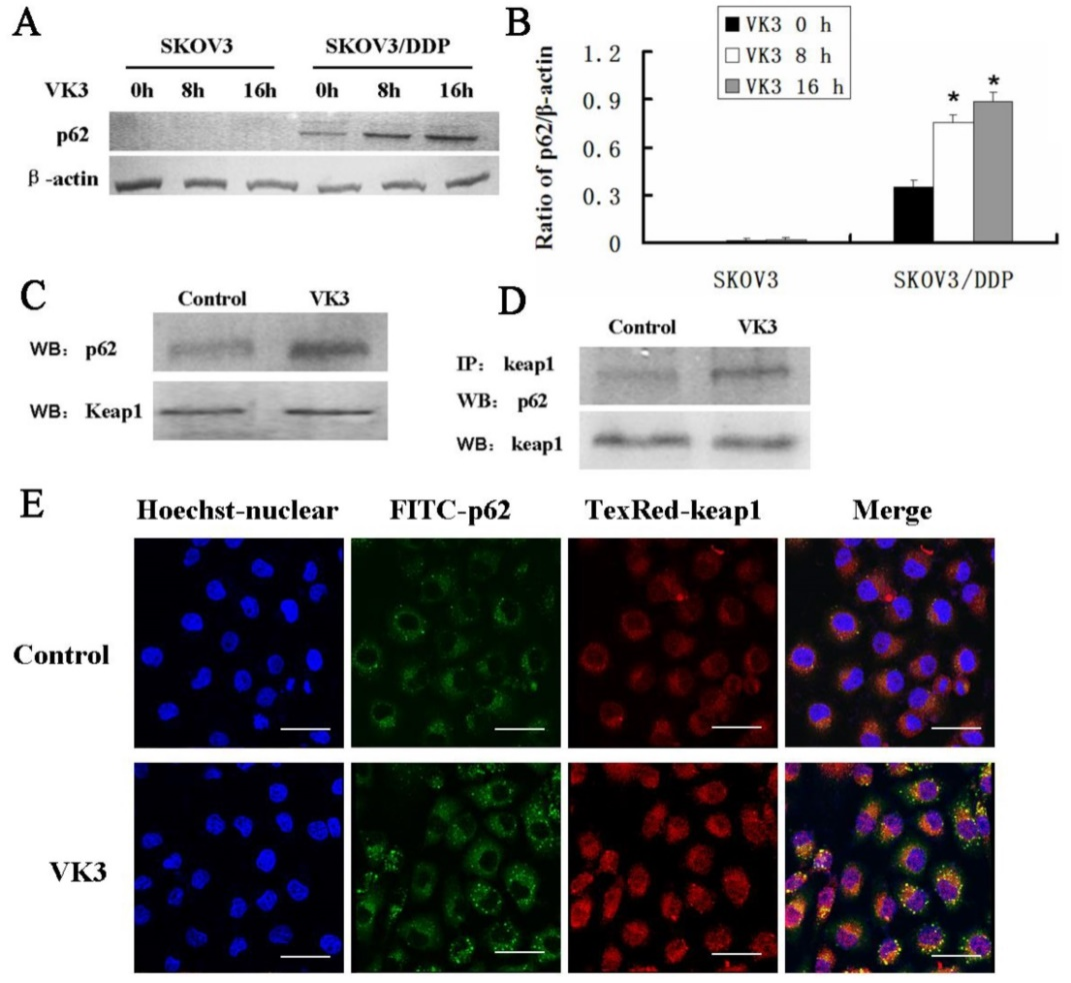
** The interaction between p62 and Keap1 increased with VK3 treatment in SKOV3/DDP cells. (A)** Both cells were treated with 15 µM VK3 for 8 or 16 h. Cell lysates were subjected to immunoblot analysis with anti-p62. **(B)** The expression of p62 in (A) was quantified. Data are presented as mean ± SD, n = 3. *P < 0.01 compared with untreated cells. **(C)** SKOV3/DDP cells were treated as before, and total p62 and Keap1 were detected by western blotting. **(D)** Cell lysates were immunoprecipitated with anti-Keap1 antibody and immunoblotting was performed with anti-p62 and anti-Keap1 antibodies. **(E)** Cells were treated with 15 µM VK3 for 8 h. Immunofluorescence of p62 and Keap1 was detected by fluorescence microscopy (scale bar, 25 µm).

Increasing evidence supported that overexpression of p62 may lead to the recruitment of Keap1 to inclusion bodies and block the interaction between Keap1 and Nrf2 7. Our results confirmed that p62 was dramatically highly expressed in SKOV3/DDP cells compared with SKOV3 cells upon VK3 treatment (Fig. 6A and 6B). Then we performed co-immunoprecipitation to analysis to determine the interaction between p62 and Keap1. The results showed VK3 increased the binding between Keap1 and p62 in SKOV3/DDP cells (Fig. 6C). Immunofluorescence results showed that p62 and Keap1 had obvious co-localization in VK3 treated SKOV3/DDP cells (Fig. 6D). Together, these results indicated that increased p62 induced by VK3 treatment activated Nrf2 through the competitive binding with Keap1.

## Discussion

**Figure 7:**
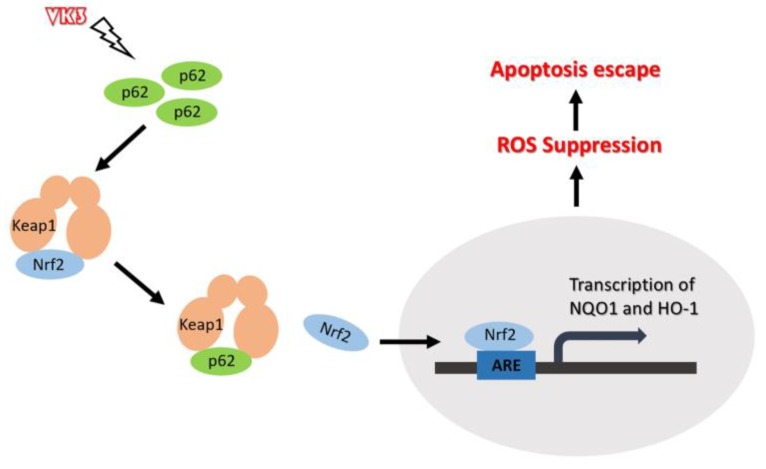
** Schematic representation of the p62- Keap1- Nrf2 pathway in ovarian cancer cells with the treatment of VK3.** Our study provides evidence that p62 promotes Nrf2 signaling through interacting with Keap1, which blocks VK3-induced apoptosis by inhibiting ROS production in SKOV3/DDP cells.

In this study, we demonstrated that highly expressed p62 in SKOV3/DDP cells activated Nrf2 through interacting with Keap1, which protect ovarian cancer cells from oxidative damage induced by VK3 (Fig. 7).

Recently, increasing evidence has shown that anti-oxidative signaling was activated in drug resistant cancer cells 30. For example, the upregulation of antioxidant enzyme catalase resulted in a 10-fold resistance to H_2_O_2_ or tert-butyl hydroperoxide in HL-60/AR leukemia cells 31. Antioxidant enzyme peroxiredoxin II overexpression inhibited cisplatin and H_2_O_2_-induced apoptosis in SNU638 cells 32. Inhibition of HO-1 by zinc protoporphyrin reduced tumor growth in gemcitabine-treated mice after pancreas carcinoma PANC-1 cell implantation 33. It's known that VK3 exhibits potent anticancer effects in various cancers such as breast, hepatic, bladder, pharyngeal, and blood cancers 34. VK3 promoted the ROS production which disrupted the intracellular calcium homeostasis, depleted cellular thiol levels, increased lipid peroxidation and finally killed the cancer cells 35. Early in 2003 it was reported that VK3 may have antitumor effect on ovarian cancer 36. In this study, the results showed that SKOV3/DDP cells (IC50 55.3 μM) were insensitive to VK3-induced cell death compared with SKOV3 cells (IC50 15.06 μM). Meanwhile, NAC experiment confirmed that the VK3 promoted the generation of ROS in SKOV3 cells. These results indicate that VK3-induced apoptosis of ovarian cancer cells and SKOV3/DDP cells are also resistant to oxidative damage caused by VK3.

Previous studies showed that Nrf2 and its downstream target genes were activated in lung cancer and epithelial ovarian cancer 37, 38. Our results showed that Nrf2 as well as the target genes HO-1 and NQO1 were overexpressed in SKOV3/DDP cells. Current studies indicated Nrf2 directly interacts with the DC domain in Keap1 through ETGE and TLG motifs of Nrf2. Furthermore, the interaction between Keap1 and Nrf2 may be disrupted in response to cellular stress, which promotes the stabilization and nucleus translocation of Nrf2 39. Previous studies demonstrated that Keap1 mutation promoted the activation of Nrf2 in several cancers such as lung cancer, carcinoma of gallbladder, and liver cancer 40, 41. Furthermore, recent studies showed that Nrf2 was over activated in platinum-resistant ovarian cancer. However, over 50% of the cancer tissues without Keap1 mutations still showed a high Nrf2 activity 38. These results indicated that more complex mechanism was involved in the regulation of aberrant Keap1/Nrf2 pathway in ovarian cancer.

Multifunctional protein p62 was original defined as the receptor of autophagy. Increasing evidence indicates that p62 also works as the signaling hub that participates in the regulation of survival pathways 42. Our previous studies indicated p62 was overexpressed in SKOV3/DDP cells which were resistant to cisplatin, suggesting that p62 may protect cell from oxidative damage caused by cisplatin 43. In this study, Nrf2 as well as its downstream genes HO-1 and NQO1 were upregulated in SKOV3/DDP ovarian cells as a result of oxidative damage caused by VK3. Furthermore, Nrf2 nucleus location was suppressed while we inhibited p62 expression in SKOV3/DDP cells, which increased the cell sensitivity to VK3-induced apoptosis. Currently, p62 was shown to interact with DC pockets of Keap1 through STGE motif, which increased the dissociation of Nrf2 from Keap1 complex 23. Our studies demonstrated that overexpressed p62 was co-localized with Keap1 in SKOV3/DDP cells after VK3 treatment. These results indicated overexpressed p62 activated Nrf2 signaling through binding with Keap1 and inhibited ROS induced by VK3. To further confirm the anti-tumor effects of VK3 and the role of p62-Keap1-Nrf2 signaling in ovarian cancer, experiments *in vivo* is needed in the following study. Interestingly, our previous studies have proved that p62-mediated selective autophagy was involved the survival mechanism of SKOV3/DDP cells through increasing the degradation of damaged proteins. Considered the role of p62 in autophagy, our future studies will focus on the regulation between Keap1-Nrf2 anti-oxidative pathway and autophagy with VK3 treatment.

## Conclusion

In summary, our study found that p62 activated Nrf2 signaling in SKOV3/DDP cells through its interaction with Keap1 conferred resistance of SKOV3/DDP cells to VK3. Notably, inhibition of p62 expression suppressed Nrf2 signaling which promoted cell death induced by VK3. These findings increase understanding in the resistant mechanisms of oxidative damage caused by chemotherapy and provide critical therapeutic strategy of ovarian cancer by targeting p62 to enhance oxidative damage.

## Abbreviations

VK3: vitamin K3
HO-1: heme oxygenase 1
NQO1: NAD (P)H quinone oxidoreductase 1
Nrf2: Nucleus factor erythroid-2 related factor 2
Keap1: Kelch-like ECH-associated protein 1
ROS: reactive oxygen species
NAC: N-acetylcysteine
